# Reply to formal comment on Griffiths *et al*. (2017) submitted by Gajewski (2020)

**DOI:** 10.1371/journal.pone.0254481

**Published:** 2021-08-03

**Authors:** Katherine Griffiths, Neal Michelutti, Marianne S. V. Douglas, John P. Smol

**Affiliations:** Paleoecological Environmental Assessment and Research Laboratory (PEARL), Department of Biology, Queen’s University, Kingston, Ontario, Canada; Universidade de Vigo, SPAIN

## Abstract

Gajewski offers a formal comment on Griffiths *et al*. (2017), a paper that explored how microclimates and their varying ice cover regimes on lakes and ponds in Arctic regions modified the diatom assemblage responses to recent warming. One of Gajewski’s main criticisms is that the microclimate classification scheme used in Griffiths *et al*. (2017) is merely anecdotal; a claim which ignores the value of observational evidence and misunderstands the frequency that each site was visited or surveyed. We clarify that the study sites were visited multiple times via recurrent aerial surveys and ground observations dating back to the 1970s, which supports our microclimate classification scheme. Many of Gajewski’s claims regarding climate, catchment characteristics, and ice melting properties from field locations he has not visited were refuted by veteran Arctic scientists with long-term field experience in these regions. In addition, Gajewski makes several criticisms concerning radioisotopic dating, core chronology, sediment mixing, diagenesis, and preservation of bioindicators that relate more to general paleolimnological assumptions than to conclusions reached by Griffiths *et al*. (2017). Research from the 1980s and 1990s, when scientific consensus on these issues was first reached, readily show that the methodologies and data interpretation of Griffiths *et al*. (2017) are sound. We appreciate the opportunity to expound on the finer details of the Griffiths *et al*. (2017) paper, work based on field research by the study’s co-authors spanning almost three decades, with additional observations from colleagues dating back to the 1970s. We address Gajewski’s criticisms with relevant literature, expert statements, and a few clarifying comments.

## Introduction

In our study [1] we examined sediment cores from lakes and ponds in the Canadian High Arctic to explore how fossil diatom assemblages at sites with different microclimates and ice phenologies responded to regional warming over the past ~200 years. Our paper was a culmination of over 35 years of work conducted by us and others on the Cape Herschel ponds and nearby areas. Briefly, we posited that the timing and degree of change in the diatom sedimentary assemblage can be modulated by the microclimate where, although all the sites should have experienced similar overall trajectories of regional warming, the diatom composition responded differently based on the microclimate category and other site-specific factors. The mechanism we suggested is that the diatom assemblages were indirectly driven by variations in microclimate associated with the length of the ice-free season. Recent regional air temperature increases have lengthened ice-free seasons and, in some sites, allowed for the establishment of extensive aquatic mosses (i.e., habitat for epiphytic diatoms) and other periphytic habitats. This resulted in a shift in diatom composition from pioneering taxa that thrived in cool conditions with short growing seasons to more diverse assemblages with complex growth forms (stalked and tube-dwelling species, many of which take advantage of newly established vegetation microhabitats). The significance of our work was to suggest a mechanism by which a commonly identified pattern of diatom assemblage changes may arise in response to recent regional warming, and to be a note of caution in the interpretation of regional patterns from a single location. For example, a diatom record that registers a shift to more diverse and complex assemblages in the 1980s, may not necessarily be a signal that this is the onset of regional warming. Rather, it suggests that the ice-free period has lengthened to a degree that allowed complex diatom forms to establish at that site at this time.

Below we systematically address the three main points of contention outlined in Gajewski’s comment.

## Criticism 1. Gajewski questions the characterization of our sites into different microclimatic classes

Specifically, he notes, “*Griffiths et al*. *2017 divide their sites into four classes—“warm”*, *“cool”*, *“cold”*, *and “oasis”- and this is the basis of all interpretation*, *however they have insufficient data on ice cover*, *and do not consider other factors*, *such as lake depth and nutrients*.”

### Division of the sites

Our limnological monitoring program at Cape Herschel has generated some of the longest (if not *the* longest) semi-continuous datasets from lakes and ponds in the High Arctic. These data are highly suitable to answer the questions we posed. Given that long-term lake water thermistor data and site-specific air temperatures were not available, we categorized the study sites based largely on long-term observational records from multiple mid-summer sampling campaigns and on our observations spanning nearly three decades. The microclimate differences we describe were obvious in every field season and were further supplemented by similar ice-cover observations collected by Dr. Wes Blake, Jr, and his colleagues, dating back to the mid-1970s, as described and documented below.

Gajewski is mistaken when he interpreted the dates listed in Table 1 of our paper as the only times we visited or observed ice cover on the ponds. Rather, what was presented in our article (as Table 1, [1]) are the notes taken only from days that detailed water chemistry sampling occurred (up to 58 water chemistry variables from each site as index values for these ponds and lakes). These were usually taken once a season, although more often for some lakes. We reiterate that the sites were visited repeatedly over each field season from 1983–2011 (usually lasting two weeks, and for several months in 1986, spanning from thaw to freeze). Furthermore, the lower Elison Pass area of Cape Herschel is visible from our base camp whereas the outlet from Moraine Pond was our drinking water supply, and so we visited that pond nearly every day during the field season. Snow/ice patterns were noted for all the study ponds during our many other excursions to various points on Cape Herschel and during regular fly-overs of many of the sites (including nearby Pim Island).

Peer-reviewed research has shown the power of observational evidence from field surveys (in combination with other lines of evidence) to document, for example, changes to ice cover [2]. The personal experiences of two of our co-authors should not be devalued. John Smol has visited Cape Herschel (and nearby Pim Island almost as often) regularly, nearly every 3–5 years for the past 28 years together with Marianne Douglas, who has likewise visited these sites for over two decades. Prior to that, Dr. Weston Blake Jr., who was emeritus scientist at the Geological Survey of Canada, performed decades of repeated observations and sampling of these lakes. Although not discussed in our paper, it was Blake (whose written comments on ice melt are discussed further below) who first pointed out that the ice covers between the sites were markedly different and directed the research team in 1983 to visit Col Pond first as, at that point in the summer, it would be the only pond that was ice-free. Our division of the sites at Cape Herschel and Pim Island is based on ground-truthing from decades of observations by the paper’s co-authors, and other members of the Cape Herschel Project.

In 1986, the sampling season at Cape Herschel spanned the entire growing season (June-August; thaw to start of re-freeze) such that for four of the 10 sites (Col Pond, Elison Lake, Moraine Pond, and Plateau Pond 2) we documented the timing of ice-off and -on. Cape Herschel and Pim Island were also visited early in the season in 1983 (providing a context for early season ice cover at West Lake) and Proteus Lake and Paradise Pond were added to the sampling regime in 1987, when the lakes were sampled in late summer. Consequently, for seven of our ten sites we have convincing observational data of the early and late season conditions with relative dates of ice-on and, to a slightly lesser extent, ice-off.

High Lake was not directly sampled until 2011 (but we, and Blake before us in the 1970s, had repeat observations of ice and snow cover from flyovers in helicopters) as it never had open water in all of the years when Pim Island was sampled. As such, High Lake is inferred to be one of our coldest sites, with one of the shortest ice-free seasons. Given the start of winter is mid/late August [3], it is reasonable to infer that the ice-free season at this lake is relatively short, as the ice does not start melting by mid-July while all the other nearby sites are partially or completely ice-free. By contrast, Elison Lake currently has an ice-free period of ~3 months. Sverdrup Pass, where the other two “oasis” ponds are located, was only visited by our team twice in mid-summer, in the 1980s and in 2011; however, these ponds are located in a warmer climatic sub-region (see below, studied by colleagues), and consequently experience a longer ice-free season.

Our detailed observations are supplemented by those of previous researchers who have worked extensively on Cape Herschel and nearby Pim Island, such as the late Dr. Weston Blake Jr. (1930–2021). In a written communication asking him to comment on ice cover changes in the area, he noted:

“*During my field seasons at Cape Herschel and Pim Island*, *which date back to the 1970s*, *the Plateau area has always been colder and more ice covered*. *The Col always opened first*. *The lower Cape area was always warmer and less ice covered*. *The top of Pim Island was of course always colder and more ice covered given its location and elevation*”.

Signed by Weston Blake Jr. on Sept 19, 2019.

### The “oasis” sites

Gajewski criticizes the characterization of “oasis” sites in Sverdrup Pass (which we only had the opportunity to visit and sample twice, in 1984 and once in 2011), which he argues is not warmer than Cape Herschel. However, many other researchers have worked in Sverdrup Pass (e.g., [4]), specifically because it has consistently been recognized as an Arctic oasis with anomalously warm temperatures (e.g., [5]).

Sverdrup Pass is in a different ecozone than Cape Herschel and Pim Island (Northern Arctic and Arctic Cordillera respectively; [6]). Cape Herschel and Pim Island are situated in the cold, sea-ice-filled waters in Smith Sound of the Nares Strait and experience a more ice-dominated climate than the continental Eureka/Nansen Sound and its adjacent lowlands (in which Sverdrup Pass is included). The 1950s-1970s provided some of the greatest detailed climate data for the region, while currently there are only three meteorological stations for Ellesmere Island. Using weather station networks and measurements spanning the years from 1953 to 1972, the Canadian Arctic Islands are subdivided into different climate regions based upon the major climate controls (such as cyclonic activity, sea-ice water regime, broad-scale physiographic features, and net radiation) [3]. These climate regions are further sectioned based on major local variations in local topography, aviation weather, maritime influence, temperature, precipitation, snow cover, and wind. For example, compared to Nansen Sound and its adjacent lowlands (including Sverdrup Pass), the Nares Strait region (including Cape Herschel and Pim Island) is characterized as having greater cloud cover, less July irradiance, later stream break-up, cooler July temperatures, and an earlier start to winter (by 5–10 days) and a later end to winter (by 5–10 days) [3]. These seemingly small differences (up to 20 days/year) can appreciably affect the length of an already short “summer” season (i.e., when the melt season is 90 days or less, an extra 10–20 days can make a substantial difference).

Gajewski presents temperature data from field camp reports at Sverdrup Pass and Cape Herschel over the course of the years 1974–1993. He admits, “*there is little overlap between dates where Sverdrup Pass camps reported and when Herschel did*.*”* The variability in climate among years is simply too great for making direct comparisons, overlapping years are required. Despite these obvious shortcomings, the data Gajewski presents run counter to his own arguments. In fact, Gajewski states that “*June tends to be warmer in Sverdrup Pass than on in* [*sic*] *the Herschel area*” and that “*Below zero temperatures seem to return in late August at approximately the same time”* (and seemingly slightly later at Sverdrup Pass), thereby inadvertently confirming our classification scheme. Importantly, it is the early temperatures that are critical for ice melt. After presenting these data, Gajewski concludes that “*there are no data in May and early June to compare the end of the ice-cover season*”. The lack of data spanning this critical period from ice-on to ice-off is exactly why we had to rely on our three decades of observations. In summary, Gajewski presents temperature data, that we did not use, only to argue that these data cannot be used to prove our classification scheme. Moreover, even if accepted, his analysis suggests that the critical early melt period (May and early June) may be warmer at Sverdrup Pass, as it is already warmer by mid-June.

Additionally, Gajewski asserts that Sverdrup Pass is vegetated because of substrate type and glacial meltwater, and that the greater vegetation in Sverdrup Pass is not a reflection of the warmer climate. However, in a study of vegetation and climate of the Queen Elizabeth Islands the Sverdrup Pass region was consigned to a completely unique, warmer bioclimatic zone that supported a denser and more diverse vascular plant community, a division that could not be explained simply by accounting for substrate type or moisture regime [5]. It was observed that “*The mountains of Axel Heiberg and Ellesmere islands create a barrier that effectively shelters an intermontane region from both the central Arctic Ocean climate and travelling cyclonic systems*. *In this large intermontane zone regional minimums of cloud cover and maximums of temperatures and melt season duration are found*.” [5]. Using meteorological data from permanent weather stations and non-standard weather data (seasonal scientific and oil-industry camps), the Eureka Sound intermontane area, which extends into the Sverdrup Pass region, was identified as having a melt period that averages “*15–20 days longer than in coastal regions adjacent to the polar pack-ice*”, and as unlikely (as compared to the coastal regions) to “*experience mean daily temperatures below freezing once the melt season is established*” [5]. Also, this intermontane region, due to the rain-shadow effect, experiences the lowest annual precipitation of all regions of the Queen Elizabeth Island [5].

In summary, while long-term comparative historical temperature data do not exist for these remote regions (which is why we conduct paleoecological analyses), there are repeat observations and visits of our study sites by our co-authors and other Arctic researchers spanning decades that support our microclimate divisions. It is reasonable to assume that the geological factors that partially differentiate the microclimatic differences among our sites (e.g., altitudinal differences, proximity to the coast) at present day were similar to conditions ~200 years ago (our period of interest) as the region has not experienced any substantial tectonic movement, changes in air-mass movement, or isostatic uplift over such a geologically short timeframe. This is not the case when considering Holocene scales (see section 3).

### Nutrients

Gawjeski speculates, without providing any evidence, that “*another interpretation of the “oasis” sites is that they are highly productive due to continual water supply from nearby glaciers and combined with bedrock*, *this provides more available nutrients*”. Gajewski’s speculation, however, is not true. We can confirm that the oasis sites are not connected to any continuous sources of water.

Additionally, Gajewski regularly refers to the oasis sites as “*highly productive”*. The Sverdrup ponds are far from being highly productive. Lake and pond water chlorophyll *a* analyses are the standard measures for estimating lake or pondwater biomass (standing crop) or productivity. Both Sverdrup ponds are so unproductive that they are below the detection limit of analyses for chlorophyll *a* (corrected for phaeophytin) at < 0.1 μg/L and uncorrected at 0.5 and 0.6 μg/L for Sverdrup ponds 5 and 8, respectively (i.e., amongst the lowest chlorophyll *a* values one can find in North America [7]). Therefore, Gajewski’s subsequent comments regarding the supposedly “highly productive” nature of Sverdrup ponds, are incorrect. Yes, overall annual production (the operative word being “annual”) would be relatively higher in the warmer Arctic oasis like Sverdrup Pass due to the longer growing season and reduced ice cover (consistent with our data, findings, and interpretations), but water-column production is low at these sites, opposite to Gajewski’s conjectures.

While it is true that the “oasis” sites generally have higher nutrient concentrations compared to most of the other ponds in this study (as warmer sites would be expected to, as nutrient release from the catchment is accelerated), there is one important exception—the “cool” site, Moraine Pond. Moraine Pond has very similar total phosphorus (TP) concentrations to the “oasis” sites (13.6 μg/L vs 15.8 μg/L and 13.9 μg/L for SV5 and SV8 respectively) due to its relatively large catchment for its size. However, the Moraine Pond sediment record still registered a climate-related diatom assemblage shift with decreasing relative abundances of commonly epipelic diatoms and increasing proportions of epiphytic taxa. In contrast, Sverdrup Pass’s SV5 and SV8 recorded different assemblage shifts, namely changes among epiphytic taxa. These observations are not consistent with Gajewski’s assertions that nutrients are the driving factor at these sites.

In addition to our study sites, there are other, high-nutrient lakes and ponds across the Arctic (although they are moderately rare) that have shown the same transition from largely epipelic and epilithic diatom assemblages to higher diversity assemblages with increasing proportions of complex growth forms including epiphytic taxa. For example, Self Pond (Ellesmere Island) in the High Arctic, shows an unambiguous transition from epipelic to epiphytic taxa and has TP concentrations that are very similar to the “oasis” ponds of 12.1 μg/L [8], or pond MV-AT on Melville Island, showing the same type of diatom compositional transition and again with a TP similar to the “oasis” ponds (12.7 μg/L) [9]. What is consistent amongst these diverse ponds is recent warming.

In contrast, though, there are other high-nutrient sites in the Arctic that have quite short ice-free seasons. Meretta Lake, on Cornwallis Island, for example, has had a relatively short ice-free season. When limnologists studied Meretta Lake during the IBP Ecosystem Study, the lake became ice-free for only a few weeks in 1969 and 1970, and never lost its central ice cover in 1971 (see Fig 4 in [10]). However, raw sewage was dumped into this lake for nearly 50 years, starting in the 1940s, leading to high nutrient influxes. Despite this, there was only a very muted response in the diatom record of the lake sediment core [11]. Meretta Lake’s assemblages are dominated by small fragilarioid taxa [12], epipelic generalist and opportunistic taxa that tend to thrive in a diversity of conditions where other diatom species struggle. Yet, despite an influx of nutrients to the site, the sedimentary diatom assemblage did not undergo a marked shift towards a greater diversity of epiphytic forms.

Another example is Annak Lake [13–17], a lake also used for sewage discharge since the 1960s by the community at Sanikiluaq situated on the Belcher Islands (Nunavut). It has a well dated ^210^Pb profile, that is further constrained by stable geochemical metals and other proxies linked to human sewage in the core profile, anchoring and corroborating the ^210^Pb dates (as described in the cited papers). Modelled TP concentrations show hypereutrophic conditions (TP > 600 μg/L) and elevated nutrient levels in Annak Lake, yet the diatom record shows that, while the lake remained ice-covered largely in the 1960s, the diatom assemblage was overwhelmingly dominated by small fragilarioid taxa. It was only later (during the two decades following the sewage inputs), but coincident with warming and declining ice covers, that the assemblage changed, reflecting the establishment of new aquatic habitats and longer growing seasons.

Nutrients, due to the greening of the Arctic and increased soil development and erosion, can play a role in some of the diatom assemblage changes we recorded (and we address this in our paper–see page 17, bottom paragraph [1])–and this is most likely why we saw an increase in *Nitzschia* species (a nutrient indicator in Arctic lakes and ponds) in many of our sites. But this is different than the shift in diatom life strategy from commonly epipelic/epilithic taxa to commonly epiphytic species and more complex growth forms requiring longer open-water periods. This particular compositional change was detailed in our publication as a shift from “condition 1” to “condition 2” and is characterized by relative decreases in commonly epilithic and epipelic species to increasing relative abundances of commonly epiphytic species (a major habitat and life strategy shift). We went into detail on how that mechanism might work (i.e., how changes in the duration of ice cover might have an effect on the establishment and growth of aquatic mosses and how those mosses support a diverse array of diatoms) throughout our paper, but especially in the section “Mechanisms of change”. The Gajewski comment fails to consider the biology of the diatom taxa. Rather, the taxonomic information of our diatom records was reduced to principal component axes, without understanding the autecology of the species considered. For example, our cores from West Lake do register diatom compositional changes (which we discuss in our publication, and which is addressed further below), but these changes do not reflect the establishment of novel epiphytic habitats, which is the ecological change we are tracking. Such an interpretation requires an understanding diatom autecology. A shift among diatom taxa with similar affinities/tolerances to prolonged ice conditions does indeed signal a limnological change, but not a change in ice cover resulting in the opening of novel habitats. An understanding of the autecology of the diatom taxa is missing from Gajewski’s analysis. This understanding is fundamental in interpreting the diatom data.

Additionally, subfossil chironomids, an independent bioindicator group that respond to temperature and habitat changes, but that do not respond directly to nutrients [18–20], were analyzed from our “warm” sites [21], using the same sediments as a previous study of diatoms on the “warm” Cape Herschel ponds [22]. The chironomids showed striking changes indicative of increased diversity and increases in macrophyte-associated taxa, matching the diatom data and the inference of aquatic moss establishment in these ponds. Two independent lines of evidence based on organisms from different phyla support the same conclusion.

### Depth

Gajewski notes that “*the four groups of lakes differ in depth and nutrient concentration which would affect diatom abundance and diversity*”. Ideally, we would have liked to have sampled the shallow, elevated, exposed ponds on Pim Island as our “cold” sites. Unfortunately, due to more extensive ice conditions on Pim Island, very few sites exist, and none of these sites possessed depositional areas that would allow us to obtain suitable sediment cores because of their low productivity, small size, shallow nature, and extremely small catchments (much smaller than any of the Cape Herschel ponds). However, our study sites are still comparable. In particular, High “Lake” (a “cold” site), although originally called a “lake” by Blake in his early field notes, is ecologically and physically a “pond” (i.e., defined here as freezing to the bottom in winter), and comparable in size and depth to Elison Lake (a “warm” site), which is also ecologically a pond. High Lake is only 4 m deep at its centre and Blake attempted to obtain a sediment core through the ice in June 1981. Based on his experiences with the site, Blake suggested that we should consider it a “cold” pond when sampling, given the ice thicknesses that can develop on nearby lakes. For example, a nearby lake on Ellesmere Island had 5.45 m of ice in June of 1986 and in August 1987, still had 3–4 m of ice [23]. Field notes from June 1981 documented ice thicknesses or both West Lake and 260 m Lake on Pim Island of 2.4 m and 2.6 m, respectively. Ice thicknesses from Ward Hunt Lake off the northern coast of Ellesmere Island recorded ice thicknesses that “ranged from 4.0–4.1 m when measured in May 1998 and August 2003, with a moat of open water that develops over the littoral zone of the lake in late summer” [24]. Although we are not able to sample in winter, it is highly likely that similar ice thicknesses can develop on Pim Island, and that likely all, or almost all, of High Lake freezes to the bottom and therefore can ecologically be classified as a “pond”, despite its informal name.

The other two “cold” sites in our study are ecologically classified as lakes and therefore tend to form several meters of ice over the winter (see above) but do not freeze completely to the bottom (although the nearshore littoral would do so). This thick layer of ice on lakes is retained even longer through the summer season than nearby ponds. The shift that we are tracking from a depauperate assemblage dominated by a few epipelic taxa to an assemblage with a greater diversity of benthic life strategies has been well documented and reported on in both lakes and ponds across the Arctic (e.g., [25, 26]).

All diatoms found in the lake sediment cores were benthic taxa with the one expected exception: the planktonic diatom *Cyclotella radiosa* in the core from West Lake, our deepest site. Therefore, almost all of the diatom production in our study is benthic and would respond to benthic processes. Aquatic mosses grow very slowly in the Arctic and can be up to 10 years old [27]. They can establish in Arctic lakes and ponds (e.g., [28, 29]), and their growth is limited primarily by snow and ice cover (i.e., they do not grow when it is completely dark and/or if they are frozen) but also by underwater irradiance, which is affected by the length of the day and the sun’s elevation. The length of ice-free conditions (i.e., conditions with sufficient light for photosynthesis) is critical, regardless of depth–open water, not snow-covered ice, are required for littoral mosses (and for diatom epiphytes) to grow. Consequently, the mechanism that we are investigating is as applicable in the lakes as it is in the shallower ponds.

### Ice melt phenology

Gajewski argues that the melting candled ice for Arctic lakes (taken from observations of primarily Alaskan lakes [30]) would scour and disturb nearshore sediments and vegetation. This Alaskan lake setting that would not be reflective of the environment sampled on Pim Island or other High Arctic sites. In reality, many Arctic lakes support aquatic mosses and nearshore sediment (e.g., [29]). Our co-authors have witnessed ice melt at Cape Herschel and other High Arctic settings for decades and have repeatedly observed extensive, mature, and clearly intact moss banks emerge from the thawing ice year after year in our study ponds and in other lakes across the Arctic. The fronds of the mosses in many of these ponds and lakes are often well over 6 cm long, and frequently longer, with mosses of similar size in a High Arctic setting reaching ages of 6–10 years (e.g., [27]). Presumably Gajewski is assuming that, after scouring each year, new mosses would have time to grow and establish in the very short summer; however, given bryophyte life history and physiology, Gajewski’s hypothesis is untenable given the size of the mosses established in many of these lakes and ponds. It is possible that ice scour (but not due to candling) could indeed affect moss growth, but this has only been observed on larger water bodies whereby the wind can push a floating ice “pad” against the shore, resulting in moss disturbance limited to the immediate nearshore environment (for example at Char Lake, Cornwallis Island). However, that does not preclude the establishment of mosses at these sites (as in Char Lake; [25, 27]) as in many of these lakes one can see moss bands that start below the area of ice scour and continue to the limit of the photic zone.

It appears as if Gajewski is arguing, in a general sense, that ice melt processes can disturb nearshore sediments? Yes, of course, this could happen. We respectfully question the relevance of Gajewski’s comment to our study cores that: 1) show near-monotonic declines in excess ^210^Pb activity to background levels; and 2) abrupt transitions in diatom assemblages that in some cases show almost 100% species turnover. Neither of these are possible if ice melt processes disturbed the sediments of our study ponds.

Gajewski’s comments on ice melting in lakes also lack the environmental context that influences ice phenology in these extreme climates, such as the high elevation lakes on Pim Island. As noted above, ice on some of these High Arctic lakes can be 3–5 m thick in August, with only shallow nearshore moats of open water at the height of summer thawing. Gajewski cites Hobbie [30] to support his contention that 80% of a High Arctic lake’s ice cover can thaw in 24 hours.

When asked about this contention, the chapter author John Hobbie (written communication, July 26, 2020) states that:

“*The 1984 publication was based on detailed observations on a deep lake in the Brooks Range of northern Alaska*. *A 2 m-thick ice cover melted down to less than 0*.*5 m thick over 1 month*. *At this point the ice crystals separated and floated vertically*. *A slight breeze often set hundreds of square meters free; they immediately fell on their sides and melted within hours*. *Finally*, *a strong wind pushed the whole ice sheet towards the north end of the lake at which time all the ice crystals melted in one day*. *Thus*, *a sheet of very thin ice that had covered 80% of the lake disappeared in a single day*. *In this same publication a study of a lake in northern Greenland is described; a 3*.*4 m thickness of ice was reduced to 2*.*0 m in a single summer so there was no loss in area but a sizeable loss in volume*. *Lakes on Pim Island would be like the Greenland lake and would not thaw in 24 hours*”.

Furthermore, the observations on Alaskan lakes in [30] were clearly important work but were not meant to be applied so broadly. Gajewski fails to acknowledge that sediment accumulation persists in the nearshore of these lakes and it is therefore not plausible that an entirely new population of nearshore vegetation is re-established every year. Also, if ice development were to preclude moss growth, why then are some of the shallowest ponds on Cape Herschel 100% moss covered (repeated personal observations). We have watched waterbodies lose their ice, repeatedly, over decades. If mosses were destroyed by ice each year, the massive moss beds (often several decades old) that carpet the bottom of these ponds and lakes could not exist.

### Criticism 2. Gajewski challenges our results based on dating of the study cores

Specifically, he notes “*Problems with their chronologies and lack of dating in some cores make it difficult to test hypotheses about the timing of changes in the diatom assemblages*”

Dating is notoriously difficult in the Arctic due to very low rates and concentrations of ^210^Pb deposition and is a well-known and documented challenge in Arctic paleolimnology [31]. Therefore, we were conservative in our interpretations based on dates. For our “warm” sites we stratigraphically matched our cores to previously dated sediment cores from the same ponds [22] using biostratigraphic boundaries clearly delineated by the diatoms (i.e., the occurrence of almost 100% species turnover), a commonly utilized and accepted method not only in paleolimnology but also in paleoceanography and geology, and which is the basis for the worldwide correlation of the geological timescale. The diatom boundaries in the previous study [22] occurred in the cores at approximately equilibrium depth (the point where supported fraction of ^210^Pb met the unsupported fraction)–which was ~ 1850 AD [22]. Gajewski’s contention is that we cannot tell that the changes occurring at the “warm” sites occurred before some of our “cool” sites. However, at Moraine Pond and Paradise Pond (both located at Cape Herschel), the diatom stratigraphic changes occurred when there was still unsupported ^210^Pb in the sediment (i.e., before equilibrium depth is reached). The depth of equilibrium is clear in our figures as we also include the activities for unsupported ^210^Pb (which is inferred (as is standard) by the activity of ^214^Bi). This shows, whether one disagrees with our chronologies or not, that the diatom changes in the “cool” sites likely occurred after the changes in the “warm” sites, i.e., after 1850 AD (as there was still unsupported ^210^Pb activity in the core at the point of transition).

Additionally, since the publication of our 2017 study, anthropogenic Hg has been analyzed in some of the same sediment cores [32] under discussion here. Due to industrialization and atmospheric deposition of Hg, the start of a post-1850 enrichment profile of Hg can be used as an independent stratigraphic marker of ~1850 AD [33]. Col Pond, Moraine Pond, SV8, and West Lake all showed marked Hg enrichment commencing with our chronological estimate for 1850 AD, while Proteus Lake also recorded a strong signal of Hg enrichment, the beginning of which (representing ~1850 AD) was within the error of the basal date of our ^210^Pb age-depth model [32]. Elison Lake also showed a post-1850 enrichment that matched the diatom stratigraphic changes (i.e., matching our 1850 AD date [22]), however the profile was muted because the magnitude is smaller, such that the timing of the trend is less clear [32]. This is unsurprising as Elison Lake is a large but shallow pond with a small catchment for its size. Paradise Pond and SV5 also had low magnitude Hg enrichment [32], which matches the low levels of ^210^Pb deposition at these sites–such that a lack of a strong trend due to low Hg deposition was foreseeable. The timing of the marked post-1850 Hg enrichment in Col Pond matched our diatom changes. An enrichment in Elison Lake, although less pronounced, matched the published chronology [22]. The same signal matching the dating in multiple cores provides considerable support for our ^210^Pb chronologies and that the diatom shifts recorded at the “cool” sites Moraine Pond and Paradise Pond occurred after the “warm” sites.

Gajewski takes issue with our basal dates for the Sverdrup Pass cores. However, the objective of our study was to examine diatom responses to recent regional warming during the past ~200 years. Thus, the sole purpose of obtaining ^14^C dates for the “oasis” cores was to ensure that the cores were *at least 200 years old*, but not to generate an age-depth model through extrapolation of the ^210^Pb dates to the one ^14^C date. We had ^210^Pb dated the SV5 core and knew that the activities were too low to obtain a reliable chronology, and so basal dates were needed. The SV8 core had not been dated yet, so as a precaution, we also sent basal sediments from that core for ^14^C dating to ensure the core was long enough (i.e., that the bottom was at least 200 years old) to answer our question. Fortunately, SV8 yielded a workable ^210^Pb chronology. This is the reason why the “oasis” cores have ^14^C basal dates, while the rest of the cores had none; the ^210^Pb dates were sufficient for the rest of the sites to demonstrate that the cores spanned our period of interest. The difference in the sedimentation rates inferred by the ^210^Pb chronologies and from the base of the last ^210^Pb dated interval to the ^14^C date are not a sufficient reason to distrust the ^210^Pb chronology or suspect these cores are less than 200 years old.

Gajewski suggests four possibilities for the discrepancy between the single ^14^C date and end of the ^210^Pb dates for the SV8 core (that there is little accumulation in the core, that the dates are too old, that there is a hiatus in the core, and that the sediment is “removed from the system” by mixing). Sediment cores are known to dewater with depth, such that intervals at the top of the core often represent shorter time intervals than the same-sized interval at depth. Therefore, many age-depth models are often curved, as is the SV8 model (e.g., [34]). A combination of dewatering and lower sedimentation rates in deeper sediments (as these sites have still experienced increases in the length of the ice-free season and increased production as a result (see chl *a* Fig)) is a plausible explanation of the discrepancy in the ^210^Pb and ^14^C dates. There is also a possibility that the basal dates are too old. The “oasis” ponds are located in dolomite landscape, and so could incorporate old carbon. To obtain the best possible dates, we sent the sediments to an expert in macrofossil retrieval and identification for ^14^C dating who isolated woody herbaceous stem fragments (SV Pond 5 and SV Pond 8), sedge *Carex* sp. achenes (SV Pond 8) and partial leaves of *Dryas integrifolia* and *Cassiope* sp. (SV Pond 5), which were analyzed for age dating. The choice of dated material should have mitigated potential of old carbon contamination. However, despite our best efforts, there is a small possibility of old carbon being incorporated into the dated material (which, of course, is true of all radiocarbon dating).

Gajewski’s third and fourth speculative possibilities for the discrepancies in the ^210^Pb and ^14^C basal dates, which were presented without supporting evidence or having observed the cores, are not likely. If there was a hiatus in the core, due to the ponds being continuously ice-covered, we would expect changes in the core lithology and in the diatoms in the lead up, which show little change through that period, especially at the high resolution at which the diatom cores were counted. The last possibility deemed by Gajewski as “likely” was that the sediments were “*constantly disturbed*, *re-suspended and removed*”. There is no evidence to support his assertions. The preservation of sedimentary profiles with curves of declining unsupported ^210^Pb indicates that sediments are, in fact, being deposited. This is further supported by anthropogenic Hg profiles for the cores [32], which would be impossible if this period of the sediment record was somehow “missing”. If all shallow ponds and lakes in the Arctic were mixed, then the hundreds of papers on ponds and shallow lakes would be invalid. Thankfully, this is not the case, as mixed cores can easily be identified by the variety of sediment proxies studied.

There are two main ways of checking whether a core has been mixed: 1) by the decay curves for ^210^Pb activities and, 2) by the stratigraphic changes (i.e., diatom or other proxy changes) in the core. Note that all our cores are sectioned and counted at very fine (0.25 cm– 0.5 cm) intervals due to the commonly low sedimentation rates of these Arctic systems. In the lakes and ponds where initial ^210^Pb concentrations were not too low, we record declines in unsupported ^210^Pb activities with depth until reaching supported ^210^Pb (background) levels (i.e., the typical ^210^Pb profile). If mixed, these profiles would be more homogeneous throughout, with very little directional trend. That is not the case with SV8. We also record similar marked anthropogenic Hg enrichment profiles [32] in these cores. Again, if these cores were mixed, we would not see evidence of Hg enrichment, but rather a more homogeneous profile. Further support for sediment mixing not being an issue comes from the striking changes we record in the fossil diatoms themselves, with marked shifts over just one or two centimeters (e.g., Col Pond, Elison Lake, Moraine Pond, Plateau Pond 2, SV5, West Lake). Some of these changes (such as at Col Pond) are nearly complete taxonomic turnovers. Even the sedimentary records with more subtle assemblage shifts (High Lake, Proteus Lake, and SV8) show some taxonomic changes (e.g., *Hygropetra balfouriana* coming in at the top 0.5 cm at High Lake that was hitherto absent), which, combined with the more marked radio-isotope profiles indicate that these sedimentary sequences are not mixed. Again, if the sediments of all shallow ponds/lakes were mixed, we would not record such striking taxonomic changes over just a few centimetres. Finally, to support that these cores have not been mixed by freeze-thaw cycles, we refer to our own observations of the sites where the cores from [22] were collected (by Blake through the ice in the 1970s). Even now, decades after the cores were taken, the imprints of the original coring holes remain in the middle of each pond, evident during flyovers in a helicopter. The impressions from the deep, meters-long cores taken by Blake through the ice remain because there is no considerable disturbance of the sediment.

### Criticism 3. Gajewski argues our interpretation of diatom changes is not consistent with long-term studies and he argues that diagenesis and mixing further complicate matters

Specifically, he notes “*The interpretation of changes in the diatoms in these short cores is contradicted by a longer sediment sequence from the same area*. *In addition*, *diagenesis or mixing of the uppermost sediments makes it difficult to draw conclusions from such extremely short sediment sequences*.”

The Gajewski criticism asserts the need for a Holocene-scale context. However, as is clear throughout our paper, our study was focused on investigating a potential driver of a commonly reported diatom response to recent regional warming (i.e., over the past ~200 years), not to climate fluctuations over the Holocene. Some of our profiles extend beyond the pre-industrial period, due to the generally slow sedimentation rate in the Arctic, but our period of interest is well-defined throughout our paper as the past ~200 years. Importantly, a Holocene context would not be appropriate for investigating the effect of microclimate differences between our sites, which would likely have experienced minimal change over the short (~200 years) timeframes; however, at millennial timescales, microclimates would likely have changed. For example, the Cape Herschel foreland only emerged from the sea ~8,500 BP, with rapid isostatic uplift occurring in the first 2,000 years, with evidence suggesting that the Cape was actually an island between 6,000–5,500 BP [35]. Some of the ponds closest to the ocean are much younger–for example “Horseshoe Pond” has a basal date of 1,740 +/- 70 (GSC-2841;[35]). The isostatic uplift was also asymmetrical [35, 36]. Additionally, regional glaciers have expanded and contracted throughout the Holocene [37–39], and ocean circulation in the Nares Strait may have been quite different, with the Nares Strait being blocked with glacial ice until 10,000 years ago [40], which would affect any interpretations one might make to infer the microclimate at these sites in the early-mid Holocene. Overall, it would be extremely tenuous to infer microclimatic differences between the sites over the entire Holocene, as the area has been so dynamic over these geologic timescales. The focus of our paper is clearly on the past ~200 years. Confusingly, Gajewski does not believe we can characterize the ponds and lakes into micro-habitats based on several decades of detailed observations yet suggests we should extend interpretations of these microclimatic differences to thousands of years ago.

#### The “cold” site–West Lake

Gajewski claims “*Griffiths et al*. *interpret the diatoms in the uppermost sediments of West Lake as showing no change in association with recent climate changes*”. This is untrue. We state: “*The diatom profile from West Lake shows no directional assemblage change*, *although it is more variable as compared to the other “cold” records (Fig 6C) with fluctuations between two epipelic species*”. Gajewski misses the point: West Lake is changing with shifts between two different epipelic taxa (taxa with the same habitat) but did not show the increase in epiphytic taxa (indicating the emergence of increasing complexity and often novel habitats) that we see at the other sites. However, it is showing dynamic changes between two commonly reported epipelic taxa with different pH optima. Poorly-buffered lakes tend to be more sensitive to climate changes than well-buffered lakes (e.g., [41–43]). West Lake is a poorly-buffered lake on Pim Island (Canadian High Arctic), and although the sediment record shows little directional change in the species assemblage (it has yet to cross the habitat thresholds related to an elongated ice free season), there is substantial variation in the high-resolution record due to climate-driven pH shifts. This is consistent with the Holocene record from this same lake [44], which showed a highly variable response reflecting climate-driven pH shifts in the diatom record.

The Holocene diatom record at West Lake was dynamic in tracking climate-driven pH changes but, importantly, we did not find increases in epiphytic taxa reflective of the emergence of novel moss habitats in West Lake. The disagreement between our shorter sediment record and the Holocene record [44] (but which is not relevant to our study) was the timing of the increase in *Cyclotella radiosa*. In our core the increase occurred at about 1800 AD, ~50 years earlier than in the Holocene record [44]. We only have four pre-1800 AD intervals (after which *C*. *radiosa* appears in moderate abundances), so the discrepancy is relatively difficult to interpret with much certainty. However, for the purposes of this reply, we will speculate as to why this may be. First, our basal date or the basal date in [44] could be incorrect. While the ^210^Pb record is amongst the best for our Arctic cores, the basal dates of any chronological sequence understandably have the greatest error. Within the error of our model, however, the oldest date estimated for this sediment interval was ~1825 AD (extrapolated assuming a constant sedimentation rate from our basal date). The Hg enrichment agrees with our dating model [32]. Second, *Cyclotella radiosa* was not responding to ice-off as was suggested [44]. Given that West Lake retains ice cover throughout the summer (until very recently), it is unlikely that West Lake experiences summer stratification, and, rather, *C*. *radiosa* may begin blooming under the ice in spring in response to light and nutrient availability with ice thinning (as seen in some planktonic taxa (e.g., [45]). However, this is just speculation based on a very few intervals of sediment of a poorly understood taxon that occurs in a section of the sediment record that is difficult to date with certainty. The uncertainty is why we ultimately decided not to include this discussion, but it is an area that may be interesting to develop for future studies.

#### Diagenesis and mixing

Gajewski’s comments on diagenesis and mixing are well-worn arguments about paleolimnology that have been asked and addressed decades ago. Gajewski’s comments are based on hypotheticals. Here we chose to focus on our actual observations and the data that we presented in our manuscript.

#### Chlorophyll a records and diatom concentrations / accumulation rates

We assess trends in primary production through the sediment cores using spectrally inferred sedimentary chlorophyll *a* (chl *a*), which includes its isomers and main diagenetic products, a moderately recent approach that can be used to reliably infer trends in the history of a single lake, but whose absolute values cannot be used to compare the histories of different lakes (reviewed in [46, 47]). The misunderstanding of the spectral chl *a* approach led Gajewski to incorrectly compare the absolute values in the sedimentary chlorophyll *a* across cores. The intended purpose of the VRS approach has always been to track *trends* in whole lake production (the emphasis on “trends” is stated explicitly in the titles and text of review papers on the spectral chl *a* approach [46, 47]).

Gajewski further asserts that we should have looked at diatom concentrations or accumulation rates. Firstly, we presented a more reliable measure of production (sedimentary chl *a*) than his proposed alternatives, one which accounts for trends in whole lake production, not just the diatoms. Moreover, diatom concentration data are fraught with difficulties [48], and in Arctic regions have been shown to be highly variable, even among cores from the same lake [49]. The only way to use concentration data is with an outstanding chronology with sufficiently refined sedimentation rates to calculate the flux–something which is rarely, if ever, possible in High Arctic cores due to low ^210^Pb deposition rates. Furthermore, if we follow Gajewski’s comments on how organic matter disappears (discussed in detail below) this would negate the use of concentration data: if the denominator (per gram dry weight of sediment) is changing in the concentration calculation due to the disappearance of organic matter over time, then the entire estimate of concentration would be incorrect.

Importantly, we in fact do have direct measurements of % carbon on cores from Elison, Col, West, Proteus, Moraine, Sverdrup 5 and Sverdrup 8, all of which show positive values refuting Gajewski’s comment that organic matter disappears [32]. Finally, the vast number of diatom ecological calibration studies are done on percentage data–no calibration sets have been successful using absolute diatom abundances. Thus, because we are linking changes to present ecology, it is rational to use percentage data.

The aforementioned reasons are why most paleolimnological studies report diatom data as relative abundances; however, we in fact do have concentration data. Diatom abundance data have been calculated for Col Pond, Elison Lake and Camp Pond [50], all of which show their highest values in the uppermost sediments (the period of recent warming), which further refutes Gajewski’s claim that “*Low diatom concentrations in sediments seem to be associated with warm temperatures at many sites*, *perhaps due to dissolution of the diatoms*” (discussed in more detail below). Biogenic silica (BSi) data from Col Pond, Elison Lake and Camp Pond [51] also demonstrate no dissolution at higher temperatures (which would be towards the top of the cores due to recent warming). Moreover, there is not always a clear association between diatom abundances and BSi, as biogenic silica also measures chrysophyte scales and cysts, siliceous protozoan plates, sponge spicules, and other biogenic silica, which is further reason for us to use trends in sediment chl *a* as our indicator of whole-lake production.

In summary, the reasons we did not assess diatom concentrations, LOI, or BSi are: 1) sedimentary chl *a* (including its main diagenetic products) is sufficiently informative (and includes all algal groups, not just diatoms) in providing a understanding of the *trends* in whole-lake production; 2) concentration data really only make sense as flux data, and Arctic dating models are not sufficiently robust for such analyses; and 3) these would not have provided any new insight into our study question (i.e., which communities are ecologically at an advantage under different conditions), making relative abundances the best measures. Nonetheless, as noted above, these data are available and refute Gajewski’s claim that there is dissolution of the diatoms at higher temperatures.

#### The “cold” sites Proteus Lake and High Lake sedimentary chlorophyll *a*

Gajewski notes that there is an increase in sedimentary chlorophyll *a* below the inferred date of 1700 AD in our sediment cores, and thus truncated from our figures. As we made clear from the abstract to the final conclusions of our paper, our objective was to investigate the response to warming over the past ~200 years. As a result, and to compare to our other records (such as Moraine Pond, PP2, and West Lake, none of which extend farther than ~200 years), we applied a cut off of ~1700 CE (extrapolated dates) for our paleo proxy records (diatoms, Hill’s N2 and chl *a*) that sufficiently covered the period of recent warming. While the High Lake paleolimnological record does not show the production increase Gajewski suggests, Proteus Lake does. It was not directly relevant to our study and the questions that we were investigating, but that did not stymie our scientific curiosity about this unusual production increase in the last intervals of the Proteus Lake sediment core. Note: this production increase does not challenge our hypothesis (see our notes on the Holocene climate and inferring microclimate based on these factors). However, we were curious, and we sent samples to radiocarbon date these basal intervals of the Proteus Lake core, in the hopes that we might be able to put these changes into context, and perhaps relate them to the changes recorded in the much more dynamic Holocene record from the nearby West Lake (which, as it is poorly buffered, we believe to be responsive to climate-driven pH shifts)–and ideally publish the results. Unfortunately, we were unable to find any datable material in the sediments at the base of the Proteus Lake core. We copy here the results from a report from PaleoTec Services, Ottawa, the expert in finding and isolating macrofossil remains for ^14^C dating to whom we sent samples and the basal samples of our “oasis” ponds (which did have datable terrestrial material):

“*Proteus Lake (PL)*, *Pim Island*:

*All five of the lake sediment samples are virtually devoid of organic material with the exception of a few moss fragments*. *While they do contain midge (Chironomidae) head capsules no other invertebrates typical of lake sediments were observed*. *Caddisfly (Trichoptera) larvae cases are present*, *but all are made of mineral grains suggesting very little organic material for case building*. *Caddisfly construct characteristic cases for protection and predation often using material available from their surroundings*. *The Proteus Lake core samples have no material available for* ^*14*^*C AMS dating*.”

(written communication, Alice Telka, June 14, 2014)

Unfortunately, having no temporal context for the earlier chl *a* increases, we were unable to make any conclusions on the Holocene climate response at Proteus Lake.

*Diagenesis and dissolution*. Some of the statements made by Gajewski regarding diagenesis are amongst the most puzzling. For example: “*Arctic sediment cores frequently have a more-organic surface layer*, *underlain by clayey inorganic grey sediment below (personal observation)*. *Griffiths et al*. *apparently interpret this as response to a longer growing season due to global warming”*. First, to be perfectly clear, nowhere in our paper do we make such an interpretation, nor do we even discuss sediment organic content. We track trends in spectrally inferred sedimentary chlorophyll *a*, which is an entirely different measure than organic content. This approach uses the light absorbance of the sediment at 650–700 nm to infer the concentration of chlorophyll *a* and its main diagenetic products, meaning we can track trends in chlorophyll-based pigments even after microbes break down the organic matter (which is the main reason that there is often less organic content at depth than in the uppermost intervals). Using this method, it is possible to track peaks in production after the recent organic matter has broken down, including deep in the core within “*clayey inorganic grey sediment*”.

Further evidence for the veracity of the spectral chl *a* method comes from a recent study, by an independent lab, which compared HPLC-derived chl *a* measurements (the current gold standard) vs VRS-derived chl *a* [52]. The authors showed that HPLC and VRS detected similar chlorophyll *a* inferences for recently dried samples. Moreover, they found that “for samples that have been stored dried at room temperature for several years there is, however, a large discrepancy between the two quantitative techniques. The VRS method is more robust with regards to degradation during storage, while HPLC results demonstrate clear storage effects.” This study demonstrates that VRS chl *a* determinations closely match HPLC measurements, and that in some instances (i.e., where long-term storage and possible pigment degradation has occurred), the VRS approach is actually favourable.

Gajewski goes on to say “*Indeed, Blake [27] has shown that in many shallow ponds, no sediment accumulates, suggesting the shallow sediments reported by Griffiths et al. could be simply a thin surface organic layer*”. This is a clear mis-reading of Blake’s paper. The 1978 progress report to which Gajewski refers was from a publication of the Geological Survey of Canada reporting on their very first attempt at testing coring equipment on Cape Herschel. As clearly noted in that document, the team was there early in the season, when Cape Herschel was entirely frozen over and snowed in, and so they did not know the precise location of the ponds. They tried coring depressions in the landscape (presumed to be ponds) at eight sites, but only five retrieved pond sediments, whilst three hit rocks. Gajewski uses this to assert that many ponds do not accumulate sediments. Once again, Dr. Blake provided a written assessment on Gajewski’s interpretation of his report:

“*Gajewski takes my 1978 paper out of context*. *We were attempting to core ponds in an exploratory way while the Cape was still frozen with snow and ice*. *We could not ascertain where we were in the depressions and so a few attempts came out without sediment as we were closer to shore and hit rocks*. *Later*, *in summer*, *we and others cored all these ponds successfully*.”

(written communication, Dr. Weston Blake Jr., Sept 19, 2019).

The sites that Gajewski asserts do not accumulate sediments are the exact same ponds to which Blake referred to above.

What were we coring if not sediment? We took several decimeters of sediment at each site, which produced typical ^210^Pb chronologies that decayed and reached background. This would not be possible if the sediment was only a recent thin surface organic layer. What we presented in our paper were standard paleolimnological techniques, including radiometric dating of sediments, fine-interval sectioning, and enumerating diatoms to assess long-term environmental change [48]. We do so in a region where dating can be difficult, but where these techniques have been applied successfully and reproducibly for decades, by many different labs, in hundreds of internationally peer-reviewed papers.

*Dissolution*. While the exact conditions that can lead to diatom dissolution are not fully understood, high salinity and high pH are the most common conditions correlated with dissolution [53, 54]. However, Gajewski states that “*Low diatom concentrations in sediments seem to be associated with warm temperatures at many sites*, *perhaps due to dissolution of the diatoms*”. This claim is incorrect and easily disproven. Temperatures, especially at levels that one might encounter in Arctic systems, have not been found to be drivers of dissolution. There is extensive literature from temperate and tropical lakes/ponds–diatom profiles recorded in lakes with water temperatures consistently over 25°C (e.g., [55]), illustrating that elevated temperatures are not in themselves a cause of dissolution. This should be evident to Gajewski, who co-authored a biogeographical study of Arctic diatom assemblages spanning a large temperature gradient from the Boothia Peninsula to northern Ellesmere Island [56] where it was concluded that “*Diatom concentrations (Conc) are not highly correlated with any measured environmental variable*” including latitude [56]. It is perplexing, then, why Gajewski believes that perceived low diatom concentrations in our sediments (concentrations that we did not report–we assessed relative abundances and production inferred by sedimentary chl *a*) are associated with temperature-driven dissolution of the valves. Diatom concentration data from Elison Lake, Camp Pond and Col Pond all record increases in diatom concentration in uppermost sediments [50], with similar trends found in the biogenic silica profiles [51] from Horseshoe Lake, Elison Lake, and Col Pond, when temperatures would be at their warmest in the past several hundred years. The data refute his claims.

Importantly, we did not see any evidence of diatom dissolution in any of our cores. Dissolution can be readily observed on the diatom cell walls including the wearing away of some of the ornamentation that can lead to the loss of smaller, lightly silicified taxa, and leaving the more thickly silicified taxa often with just the centers remaining or with porous and fragmented exterior surface (diatoms subject to dissolution have a very characteristic appearance—see plates [53]). That was not the case in any of our cores.

Additionally, the many Holocene-length fossil diatom records from Arctic regions also support the premise that temperature increases do not result in the dissolution of subfossil assemblages. This is also shown in rare instances where sediment from multiple interglacial periods are preserved. For example, a subfossil diatom record from Lake CF8 on east-central Baffin Island that captures three successive interglacial periods within the last ~200,000 years [57]. They show similar ontogenetic trends of subfossil diatom assemblages within each interglacial and no evidence of diatom dissolution despite large fluctuations in temperature over this period.

Finally, if all we are doing is simply tracking diatom dissolution, then how does Gajewski dismiss the other non-siliceous paleolimnological proxies examined in these same sites? For example, as mentioned above, chironomid fossils (which are chitinous, not siliceous) from the same cores as the initial diatom study from Cape Herschel [22] showed increases in macrophyte-associated taxa matching the diatom data [21]. This would be very unlikely if all we are seeing are the effects of diatom dissolution, suggesting dissolution is not what we are tracking in these cores.

*Ephemerality*. Gajewski states “*Interpretation of chronologies in short sediment cores from small and shallow ponds need consider sediment mixing and alteration due to drying and refilling of ephemeral ponds*”. We have already addressed his statements on mixing of the sediments, and so here focus on his argument that the Cape Herschel ponds that we analyzed are ephemeral. Of course, the ponds we cored are not ephemeral. Although some of the shallowest Arctic ponds on Cape Herschel (but not our sites), which had been permanent water bodies for millennia, are now completely drying during the longer polar summer [58], the drying up of these sites is a recent phenomenon. The drying has occurred with warming (i.e., due to increased evaporation, which was carefully documented by years of pondwater specific conductance data). Note the ponds are underlain by granite bedrock; they are not permafrost ponds. Some of the smallest and shallowest ponds began to desiccate in 2006 as warming conditions increased (due to higher evaporation leading to increasing solutes, even though precipitation generally increased). Although some of the very shallow sites on Cape Herschel have become ephemeral, paleolimnological and other data show that they were not ephemeral before 2006 (e.g., the diatom and chironomid records from Camp Pond; [21, 22])–only with prolonged warming in recent years. Importantly, this is not the case with any of our study ponds in this paper. Elison Lake, one of our “ponds” on Cape Herschel, is almost 1 km long. The amount of water that would have to evaporate in the short, cool summer is obviously not possible. Additionally, changes in the sediment diatom assemblages due to desiccation (i.e., aberrant diatom assemblages, deformed frustules), and changes in the sediment matrix (i.e., physical changes in sediment structure and texture, etc.) can be tracked within the sediment record, but we saw no evidence of this at our sites, supporting our assertion that these sites are not ephemeral. In addition, many ponds have extensive aquatic mosses–if they were ephemeral, we would not have such luxuriant aquatic growth. Finally, we have regular direct observations of these ponds spanning decades–they are clearly not ephemeral.

*Zonations*. Finally, Gajewski noted that he “*would in some instances locate the zones in different place*s” and that “*it is not always clear how Griffiths et al*. *interpret the dendrograms*”. We thank Gajewski for bringing this to our attention. The broken stick models and dendrograms included in the Dryad database were mistakenly taken from an early data exploration phase of the project (where species assignments were not finalized, and some counts were not even completed) and not the finalized dendrograms used in our manuscript. We have substituted the correct dendrograms and broken stick models for the complete dataset (which were used to create the figures in the published version of our study [1]) in the Dryad database. The zones presented in our published manuscript are based on the completed diatom counts and are correct.

## Summary

Gajewski’s criticisms of our study [1] suggest a misreading of our paper including misunderstanding the main points and scope of the manuscript. His comments disregard relevant literature and largely focus on generalities without specific reference to our data.

Below we summarize our responses to Gajewski’s main claims.

### 1) Gajewski claims the categorization of our study sites into four grouping is based on single or limited visits

The dates provided in the table were simply the dates of our detailed water chemistry analyses and were not the only times that the sites were visited or observed. Rather, many of the study ponds on Cape Herschel are visible from our base camp and even some that are not, such as Moraine Pond, were visited almost daily. Frequent aerial surveys and on-the-ground sampling, dating back to mid-1970s, support our ice cover phenologies. Our observations on ice melt, as well as those of others, extend back nearly forty years.

### 2) Gajewski argues that High Arctic ponds do not accumulate organic matter

Gajewski mis-read a progress report on the first coring attempts at Cape Herschel, when the Cape was still under snow cover and the ponds were difficult to locate [59]. All the ponds that Gajewski assumed had no organic sediment are some of the same study ponds discussed in our study [1]. He cites Blake [59] to support his claim; we provide a written statement from Blake that directly contradicts Gajewski’s interpretation. The collection of sediment cores (several decimeters in length) from the study ponds underscores the obvious–High Arctic ponds at Cape Herschel do accumulate organic matter.

### 3) Gajewski argues that meteorological data indicate no differences in temperature between Cape Herschel and Sverdrup pass

Gajewski compares temperature records from the two regions over years where there is little overlapping data, making direct comparisons impossible. Still, even with his own analysis, he shows Sverdrup Pass is likely warmer, especially at the critical spring period that determines ice melt. Gajewski, who presented these data, then concludes that these temperature data do not help resolve the issue he raised.

### 4) Gajewski argues that the chronologies in Griffiths et al. [1] are not robust enough to distinguish a difference in the timing of the diatom changes between the “warm” sites and the “cool” sites

We acknowledged in our study [1] the challenges of sediment dating in the High Arctic, which is why we were very conservative in our interpretations based on dates. The changes at the “warm” sites occurred where the equilibrium depth had been reached (i.e., where there was no longer unsupported ^210^Pb in the sediment) based on matching cores from the same ponds in a previously published study (i.e., [22]). In contrast, the diatom stratigraphic changes for the “cool” sites occurred when there was still unsupported ^210^Pb in the sediment (i.e., before equilibrium depth was reached). This suggests that the diatom changes in the “cool” sites most likely occurred after the changes in the “warm” sites, i.e., after 1850 AD (as there was still activity in the core at the point of transition). After publication, our chronologies have been independently verified with post-1850 Hg enrichment profiles [32] from the same sediment cores, which can be used as an independent stratigraphic marker of ~1850 AD.

### 5) Gajewski argues that diatom concentration, biogenic silica and loss-on-ignition data are necessary for interpretations of past change

The proxies Gajewski favors are weaker metrics of change than what we report and are often fraught with difficulties. Sedimentary chl *a* includes all algal groups (unlike biogenic silica) and the spectral method captures the main diagenetic products, which provides a consistent chlorophyll signal (and by extension a signal of whole-lake production) over long timescales much better than LOI. Concentration data are best presented as fluxes and the Arctic dating models are not robust enough for such analyses. Importantly, though, the proxies Gajewski champions would not change our interpretations. In fact, diatom concentration data [50], biogenic silica and LOI [51] (as well as % carbon; [32]) exist for several of the study cores, and these data directly contradict Gajewski’s claims. For example, diatom concentration data typically show highest concentrations in the surface sediments, which refutes his claim that warmer temperatures lead to diatom dissolution. Loss-on-ignition data similarly contradict his claim that the study ponds do not accumulate organic matter, as does the proxy of organic carbon in the sediments [32].

### 6) Gajewski argues that the trends in diatom profiles can be explained by dissolution of the valves associated with periods of warmer temperatures

If Gajewski’s assertion was correct, it would refute hundreds of papers published every year on diatom paleolimnology from subarctic, temperate, and tropical regions. His own co-authored publication [56] on diatoms from Arctic regions warmer than our study sites directly contradict his argument.

### 7) Gajewski claims that rapidly melting candled ice each spring scours High Arctic lakes and ponds of their sediment and mosses

Gajewski’s argument about ice melt is based on a study from an Alaskan lake ~2,800 km to the southwest of our study site. His incorrect application of that study to High Arctic lakes was corrected by the original author. Gajewski provides personal anecdotes on ice melt in Arctic lakes to make the claim that sediment disturbance in nearshore environments is possible. This claim is most easily refuted by the presence of multi-year moss growth along the shores and bottoms of the ponds and the fact that we collected sediment cores with marked stratigraphic profiles and / or ^210^Pb profiles with near-monotonic declines in excess ^210^Pb activities from all of the study sites. We are discussing our data, which show this is not an issue. Also, we note that we do not core at the edges of lakes and ponds.

## Concluding remarks

Our first paleolimnological study of Cape Herschel [22], arguing that fossil diatom assemblages were tracking recent warming based on changes to the duration and extent of ice cover, was perhaps understandably met with much scepticism. However, since that time, our labs have published dozens of similar papers on this topic, confirming our initial findings [60]. Likewise, independent researchers have used similar approaches to publish hundreds of papers showing how temperature-driven changes affect ice cover duration and its associated effects on biota, tracking climate change throughout the circum-polar Arctic, Antarctic, and alpine regions. Our study [1] builds on this earlier research showing how local context–the microclimate of each lake (specifically the effect that microclimate can have on the ice-free season resulting in novel substrate development)–can modify a well-described diatom response to recent warming temperatures in the High Arctic. The authors do not believe that Gajewski correctly understood our paper [1] and hope that we have adequately addressed the criticisms to our work.
